# Development of a Likelihood of Survival Scoring System for Hospitalized Equine Neonates Using Generalized Boosted Regression Modeling

**DOI:** 10.1371/journal.pone.0109212

**Published:** 2014-10-08

**Authors:** Katarzyna A. Dembek, Samuel D. Hurcombe, Michele L. Frazer, Peter R. Morresey, Ramiro E. Toribio

**Affiliations:** 1 College of Veterinary Medicine, The Ohio State University, Columbus, Ohio, United States of America; 2 Hagyard Equine Medical Institute, Lexington, Kentucky, United States of America; 3 Rood and Riddle Equine Hospital, Lexington, Kentucky, United States of America; Nazarbayev University, Kazakhstan

## Abstract

**Background:**

Medical management of critically ill equine neonates (foals) can be expensive and labor intensive. Predicting the odds of foal survival using clinical information could facilitate the decision-making process for owners and clinicians. Numerous prognostic indicators and mathematical models to predict outcome in foals have been published; however, a validated scoring method to predict survival in sick foals has not been reported. The goal of this study was to develop and validate a scoring system that can be used by clinicians to predict likelihood of survival of equine neonates based on clinical data obtained on admission.

**Methods and Results:**

Data from 339 hospitalized foals of less than four days of age admitted to three equine hospitals were included to develop the model. Thirty seven variables including historical information, physical examination and laboratory findings were analyzed by generalized boosted regression modeling (GBM) to determine which ones would be included in the survival score. Of these, six variables were retained in the final model. The weight for each variable was calculated using a generalized linear model and the probability of survival for each total score was determined. The highest (7) and the lowest (0) scores represented 97% and 3% probability of survival, respectively. Accuracy of this survival score was validated in a prospective study on data from 283 hospitalized foals from the same three hospitals. Sensitivity, specificity, positive and negative predictive values for the survival score in the prospective population were 96%, 71%, 91%, and 85%, respectively.

**Conclusions:**

The survival score developed in our study was validated in a large number of foals with a wide range of diseases and can be easily implemented using data available in most equine hospitals. GBM was a useful tool to develop the survival score. Further evaluations of this scoring system in field conditions are needed.

## Introduction

Equine neonates are highly susceptible to life-threatening conditions such as sepsis, hypoxic-ischemic encephalopathy, prematurity, and postpartum trauma that result in high mortality rates (20-60%) [1]–[3]. Treating critically ill foals is often challenging, time consuming, and expensive. Despite recent advances in equine neonatal care medicine, the mortality rate of foals with evidence of sepsis is reported to be around 30–50% [1], [4], [5]. Poor prognosis for survival, reduced ability to perform at an expected level, and financial limitations are the main reasons for which sick foals are euthanized. In many instances this is a difficult decision for both clinicians and owners. Having access to a more objective method to estimate the likelihood of survival in hospitalized foals within hours of admission could be a valuable tool. In human ICU medicine, a number of scores are used to characterize severity of disease, degree of organ dysfunction, and to predict likelihood of mortality in adults and children [6]. These include the Acute Physiology Chronic Health Evaluation II, III and IV (APACHE II, III and IV), the Simplified Acute Physiology Score II (SAPS II), the Sepsis-related Organ Failure Assessment (SOFA), and the Mortality Probability Model (MPM II) [6]–[10]. Prognostic indicators and mathematical models to predict survival in hospitalized foals have been published [3], [11]–[16]; however, to our knowledge, a validated scoring system to estimate survival in hospitalized newborn equine neonates has not been developed.

Multiple statistical methods can be used to build mathematical models to predict survival. These methods have advantages and disadvantages, and in veterinary medicine, the fact that these complex models are impractical and not prospectively validated in heterogeneous groups of foals reduces their validity and clinical use.

Boosting is a statistical tool that combines the prediction power of several models to improve the predictive performance of a final model [17], [18]. Generalized boosted model (GBM) is a type of regression model that is highly flexible and was chosen for the study presented here because of its considerable success in predictive accuracy [18]. Boosted models tend to fit data better than linear models and conclusions reached may be more credible when validated in different data sets or prospectively. In addition, there is mounting evidence that boosted models are one of the best approaches to predict outcome [17], [18].

Due to the potential clinical and financial benefits of having a survival scoring system that could be implemented in hospitalized foals within hours of admission, the goals of this study were 1) to develop a foal survival score (FSS) by means of GBM using readily available historical, clinical, and laboratory information from foals with a wide range of clinical conditions (retrospective study), and 2) to validate this scoring system in a large population of hospitalized foals (prospective study).

## Materials and Methods

### Animals

Two populations of hospitalized foals (n = 624) of less than four days of age, from three equine veterinary hospitals (The Ohio State University, Columbus, Ohio; Hagyard Equine Medical Institute, Lexington, Kentucky; Rood and Riddle Equine Hospital, Lexington, Kentucky) were included in the study. A retrospective population of foals (n = 339) from three foaling seasons was used to select variables and develop the FSS and a prospective population of foals (n = 285) from the three following foaling seasons was used to validate the performance of the FSS. This study was approved by the OSU Institutional Animal Care and Use Committee and adhered to the principles of humane treatment of animals in veterinary clinical research, as stated by the American College of Veterinary Internal Medicine and National Institutes of Health guidelines.

### Retrospective study

Prognostic variables and weights (scores) for each variable to be included in the FSS were identified by GBM and generalized linear models (GLM) in the retrospective study. Foals of less than four days of age of any breed or sex admitted to the equine hospitals were included. Foals were categorized into three groups: septic, sick non-septic (SNS) and healthy. Foals in the septic group had a sepsis score (SS) of ≥12, a positive blood culture, or both [15]. Foals in the SNS group were presented for illnesses other than sepsis (e.g. hypoxic ischemic encephalopathy, meconium impaction, failure of transfer of passive immunity or orthopedic conditions) requiring hospitalization. These foals had negative blood cultures and SS of <12 [15]. The control group consisted of 24 h old foals, presented for a routine examination of a newborn foal and classified as healthy based on physical examination, a normal complete blood count (CBC), serum biochemistry, and serum immunoglobulin G (IgG) concentrations (>800 mg/dL). The medical records were examined and information from 37 historical, clinical, and laboratory variables were retrieved (**Table 1**). These included SS, age, rectal temperature, heart rate, respiratory rate, mucous membrane color, capillary refill time (CRT), cold extremities, abnormal mentation (depression, lack of affinity for the mare, weak suckle reflux), evidence of hypoxic ischemic encephalopathy, prematurity (<320 days of gestation)[19] or prolonged gestation (>360 days)[19], abnormal foaling (dystocia, assisted delivery, C-section, premature placental separation, mare's illness), weak peripheral pulse, ≥2 infection/inflammation sites, diarrhea, packed cell volume (PCV), white blood cell count (WBC), segmented neutrophil, band neutrophil, lymphocyte, monocyte and platelet counts, total protein, albumin, L-lactate, blood urea nitrogen (BUN), creatinine, IgG, fibrinogen, sodium, potassium, chloride, anion gap, glucose, total calcium, phosphorus, and total bilirubin concentrations.

**Table 1:** Admission laboratory, clinical and historical variables categorized by outcome in neonatal foals from the retrospective study.

Variables (n = 37)	Survivors (n = 263)	Non-survivors (n = 76)	P
PCV (%)	39 (12–56)	40 (21–59)	0.39
WBC × (10^3^/µL)	8.6 (0.6–32.5)	4.3 (0.1–23)	0.01
Segmented Neutrophils × (10^3^/µL)	6 (1.9–26.5)	3.74 (0.014–30.1)	0.04
Band Neutrophils × (10^3^/µL)	0.1 (0.01–4.8)	0.28 (0.01–6.5)	0.12
Lymphocytes × (10^3^/µL)	1.2 (0.01–17)	1.0 (0.01–22)	0.21
Monocytes × (10^3^/µL)	0 (0–9.1)	0.1 (0–6.7)	0.23
Platelets × (10^3^/µL)	270 (59–552)	265 (101–618)	0.97
Fibrinogen (mg/dL)	263 (60–800)	261 (100–800)	0.72
Total protein (g/dL)	4.8 (2.8–7.7)	4.5 (3.1–6.4)	0.01
Albumin (g/dL)	2.9 (1.9–3.8)	3.1 (1.7–3.9)	0.01
L-lactate (mmol/L)	4.7 (0.8–17.9)	7 (1.17–18.9)	0.01
IgG (mg/dL)	844 (100–4000)	429 (100–1399)	0.01
Sodium (mEq/L)	138 (107–149)	137 (128–155)	0.5
Potassium (mEq/L)	3.8 (2.1–6.9)	4.1 (2–7)	0.04
Chloride (mEq/L)	99 (76–109)	95 (78–108)	0.05
Anion gap (mEq/L)	13 (4–40)	16 (7–44)	0.01
Glucose (mg/dL)	125 (10–336)	84 (5–224)	0.01
BUN (mg/dL)	20 (4–40)	24.4 (12–67)	0.01
Creatinine (mg/dL)	2.3 (0.5–22.3)	4.3 (1–17.6)	0.01
Total calcium (mg/dL)	11.4 (3.4–18.5)	11.6 (4.73–17.4)	0.34
Phosphorus (mg/dL)	5.7 (3–14.8)	6.5 (2.5–22.8)	0.01
Total bilirubin (mg/dL)	3.4 (0.8–13.3)	4.2 (1.1–14.6)	0.01
Temperature (°C)	37.7 (32.3–41.4)	37.1 (32.4–40)	0.01
Heart Rate (bpm)	100 (30–170)	100 (40–170)	0.21
Respiratory rate (bpm)	34 (12–124)	36 (18–90)	0.26
Sepsis score	7 (0–21)	13 (5–22)	0.01
Abnormal mucous membrane color	76y, 187n	44y, 32n	0.01
Prolonged CRT	83y, 180n	30y, 46n	0.19
Cold extremities	14y, 249n	36y, 40n	0.01
Hypoxic ischemic encephalopathy	36y, 227n	16y, 60n	0.11
Abnormal mentation	85y, 178n	41y, 35n	0.01
Weak peripheral pulse	6y, 257n	4y, 72n	0.17
≥2 infection/inflammation sites	28y, 235n	20y, 56n	0.01
Diarrhea	32y, 231n	8y, 68n	0.15
Prematurity/prolonged gestation	18y, 245n	20y, 56n	0.01
Abnormal foaling	72y, 191n	33y, 43n	0.01
Age (h)	24 (1–96)	11 (1–96)	0.04

Data expressed as median and range. A P value<0.05 was considered significant.

y, yes; n, no; bpm, beats/breaths per minute; h, hours.

Survival was defined as discharged alive from the hospital. Foals that died or were euthanized due to a grave medical prognosis were defined as non-survivors and included in the study. Foals euthanized for other reasons such as financial constraints were not included in the study.

### Prospective study

The same inclusion criteria used in the retrospective study were used for the prospective study. A probability of survival>50% was considered predictive of survival (positive test) and <50% of non-survival (negative test). Contingency tables were used to calculate sensitivity, specificity, positive and negative predictive values of the FSS for each season individually and for the entire population of foals included in the prospective study. Sensitivity was the number of foals accurately predicted by the FSS to survive divided by the total number of foals that survived (true-positive results). Specificity was the number of foals predicted by the FSS to die divided by the total number of foals that died (true-negative results). Positive predictive value was calculated as the number of foals that were accurately predicted to survive divided by the total number of foals that the FSS predicted to survive (true-positive plus false-positive). Negative predictive value was the number of foals accurately predicted to die divided by the total number of foals that the FSS predicted to die (true-negative plus false-negative results).

### Statistics

Data sets were tested for normality by the Shapiro-Wilk statistic and most variables were found to be not normally distributed. Medians and ranges were calculated for continuous variables. The Mann-Whitney-U test was used to compare survivor and non-survivor groups in the retrospective study. Relationships between survival and categorical variables were analyzed using contingency tables and chi-square analysis in the retrospective study. The Kruskal-Wallis statistics was used to compare SS and FSS between foaling seasons in the prospective study. Significance was set at P<0.05.

A GBM was used to determine which of the 37 variables analyzed were the best predictors of survival and to establish the cutoff values for each predictor.

GBM is a method for fitting regression models to data where certain model variables can be modify to find the best-fitting model [20]. In our study, we chose to create discrete factors from the continuous variables by selecting cutoff values that divide the population into two groups (high versus low probability for survival). We used GBM analysis to modify the cutoff values to produce the best predictive model for survival in neonatal foals.

Based on these cutoff values, variables retained in the final model were considered to be factors in the FSS: a factor was assigned a final value of 1 in the FSS if the value of the original variable was above the established cutoff value, and assigned a final value of 0 in the FSS if the value of the variable was below the cutoff value (for example: a glucose concentration of ≥80 mg/dL was assigned a value of 1 and <80 mg/dL a value of 0).

Scores (weights) for each retained variable were defined based on a GLM with a logit link run that used these factors to determine the influence of each individual variable on survival. Although the regression coefficients in the GLM were not equal we decided to use equal factors (0, 1 or 2) for the FSS to make it simple and easy to calculate.

Probability of survival was calculated for each possible total FSS. The Pearson chi-squared Goodness-of-Fit test indicated that the GLM fitted the data set well (P = 0.99). Data were analyzed with SPSS Statistics (IBM Corporation, New York, USA) and R project (www.r-project.org/) softwares [21], [22].

### Univariate logistic regression analysis for survival in the prospective study

Survival score (FSS) was categorized by two cutoff values divided into tertiles based on the distribution within the prospective population. This was then analyzed using logistic regression for binomial distribution. Crude odds ratios and 95% confidence intervals were calculated for each category.

### Receiver operating characteristic (ROC) curve

In order to determine the area under the curve (AUC) and a cutoff value above which survival could be most reliably predicted by the FSS, receiver operating characteristic (ROC) curve, sensitivity, and specificity were calculated.

## Results

### Study Populations

A total of 339 (283 hospitalized; 56 healthy) and 285 (239 hospitalized; 46 healthy) neonatal foals were included in the retrospective and prospective studies, respectively. The median age of all hospitalized foals on admission was 12 h in the retrospective and 14 h in the prospective studies (P = 0.21). In the retrospective study, 40% (114/283) of hospitalized foals were septic and 60% (169/283) were SNS, whereas in the prospective study 38% (90/239) were septic and 62% (149/239) were SNS (P = 0.58). In the retrospective study, 59% (167/283) of hospitalized foals were colts and 41% (116/283) were fillies, while in the prospective study 52% (124/239) were colts and 48% (115/239) were fillies (P = 0.11). Breeds of hospitalized foals from both studies predominantly consisted of Thoroughbreds, Standardbreds, Quarter Horse, Warmbloods, and Arabian horses. All healthy foals from both studies were Thoroughbreds.

In both studies, foals were presented for a variety of medical conditions including sepsis/septicemia, septic arthritis, omphalophlebitis, umbilical/inguinal hernia, peritonitis, meningoencephalitis, pneumonia, enteritis, enterocolitis, colic, meconium impaction, hypoxic ischemic encephalopathy, failure of transfer of passive immunity, flexural and angular limb deformities, ruptured bladder, patent urachus, neonatal isoerythrolysis, and trauma.

Positive blood cultures were obtained in 33% (38/114) of septic foals in the retrospective study and 31% (28/90) in the prospective study (P = 0.76). The median SS was eight for the retrospective and nine for the prospective study (P = 0.21). The survival rate in neonatal foals was 78% (264/339) for the retrospective and 76% (216/285) for the prospective study (P = 0.56).

### Development of the survival score (retrospective study)

Final regression coefficients are presented in **Table 2**. Six variables were retained in the final model: three categorical (cold extremities, prematurity, and ≥2 infection/inflammation sites) and three continuous (IgG, glucose, and WBC) variables. **Table 3** shows scores assigned to these variables, with a total score ranging from 0 to 7. Probabilities for survival associated with each possible survival score are presented in **Table 4**. Total scores of 0–3 represented <50% and scores of 4–7 represented>50% probabilities of survival in hospitalized foals.

**Table 2:** Generalized linear model to predict probability of survival in hospitalized foals based on data obtained from the retrospective study.

Variables	Estimate	Standard Error	Z value	P value
Intercept	−0.3072	0.55	−0.56	0.57
Cold extremities	−2.0115	0.42	−4.77	0.0001
Prematurity (<320 days)	−0.8166	0.46	−1.75	0.07
≥2 infection/inflammation sites	−0.7685	0.42	−1.8	0.07
IgG (mg/dL)	0.9877	0.35	2.8	0.005
Glucose (mg/dL)	1.1331	0.4	2.77	0.005
WBC × (10^3^/µL)	0.9043	0.4	2.22	0.02

Logit (Probability of survival)  = −0.3072 −2.0115× (Cold extremities) −0.8166× (Prematurity) −0.7685× (≥2 Infection/inflammation sites) +0.9877× (IgG) +1.1331× (Glucose) +0.9043× (WBC)

**Table 3:** Survival score in hospitalized neonatal foals*

Variables			Score
Cold extremities	no	yes	
	2	0	
Prematurity (<320 days)	no	yes	
	1	0	
≥2 infection/inflammation sites	no	yes	
	1	0	
IgG (mg/dL)	<400	≥400	
	0	1	
Glucose (mg/dL)	<80	≥80	
	0	1	
WBC × (10^3^/µL)	≤4	>4	
	0	1	
**TOTAL SCORE**			

*Note: The original scale derived from the GLM output ran from −3.5 to +3.5. The score was rescaled to range from 0 to 7; for example, Prematurity was originally coded as −1 (no) and 0 (yes). Because of this rescaling, it was necessary to calculate probability of survival as:

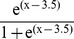
, where x is the survival score.

**Table 4:** Probability of survival for the total survival score in hospitalized foals.

Total Foal Survival Score	Probability of Survival
0	3%
1	8%
2	18%
3	38%
4	62%
5	82%
6	92%
7	97%

### Validation of the survival score in the prospective study

In the prospective study, the ability of the survival score to predict survival was determined by the association of the final and predicted outcome using a contingency table. Foals from the prospective study were classified as predicted to survive (a total survival score ≥4) or to die (survival score <4). This classification was compared to the actual outcome, of which the sensitivity, specificity, positive and negative predictive values of the FSS were 96%, 71%, 91% and 86%, respectively. There were no differences in the median SS and FSS, as well as in the survival rate between each season within the prospective study. **Table 5** shows sensitivity, specificity, predictive values, survival rates, SS, and FSS for each season, as well as all seasons combined in the prospective study.

**Table 5:** Sensitivity, specificity, positive predictive value (PPV), negative predictive value (NPV) of FSS to predict survival, and survival rate, SS, and FSS for each season in the prospective study.

Seasons	Sensitivity	Specificity	PPV	NPV	Survival Rate	SS*	FSS*
2011 (n = 83)	95%	70%	90%	85%	75%	10 (2–18)	5 (1–7)
2012 (n = 99)	96%	80%	93%	73%	73%	9 (1–26)	6 (0–7)
2013 (n = 103)	94%	72%	93%	75%	79%	9 (0–21)	6 (1–7)
**Total (n = 285)**	**96%**	**71%**	**91%**	**86%**	**76%**	**9 (0–26)**	**6 (0–7)**

SS, sepsis score; FSS, foal survival score; *median and range.

### Univariate logistic regression and ROC for FSS to predict survival in the prospective study

In the hospitalized population, foals with a FSS in the range of 4–5 (95% CI: 7.04–83.32) and 6–7 (95% CI: 36.24–228.86) were 24.2 and 91 times more likely to survive than foals with a FSS of <4, respectively (P<0.001) (**Table 6**).

**Table 6:** Univariate logistic regression for survival in hospitalized foals from the prospective study.

Variables	Range	Crude Odds Ratio for Survival	95% Confidence Interval
FSS	0–3	referent	
	4–5	24.22**	7.04–83.32
	6–7	91.07**	36.24–228.86

**P<0.01.

FSS, foals survival score.

**Figure 1:**
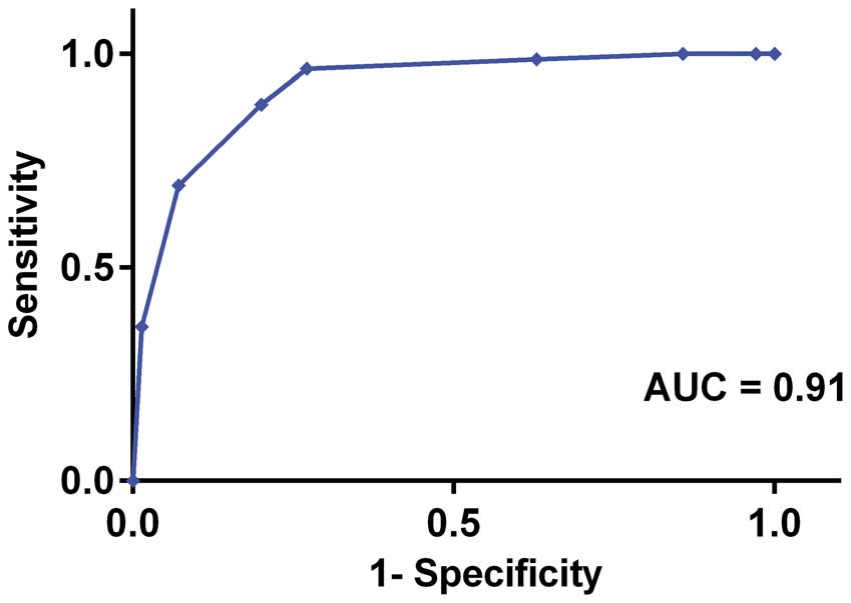
Receiver operating characteristic (ROC) curve for FSS to predict survival in the prospective study. A cutoff value of 4 for FSS maximized sensitivity (96%) and specificity (71%) to predict survival in hospitalized foals. AUC, area under the curve; FSS, foal survival score.

The AUC determined by ROC was 0.91 (**Figure 1**). The optimum cutoff value was defined as the point where the numerical value of sensitivity and specificity was maximized. ROC analysis indicated that an admission FSS of 4 for prediction of survival had a sensitivity of 96% and a specificity of 71%.

## Discussion

Caring for critically ill foals is often demanding and expensive, and having access to a scoring system to predict survival shortly after admission can be a valuable tool to clients and veterinarians. In our study, we used readily available clinical, historical, and laboratory information from a large and heterogeneous population of foals admitted to three equine hospitals to develop such a scoring system. Subsequently, the FSS was validated in a prospective study that included foals from three foaling seasons with similar results among seasons. A high FSS was associated with increased odds for survival.

A number of regression models to predict mortality in hospitalized neonatal foals have been developed; however, variables retained in the final model differ among studies, reflecting different study populations and experimental designs [11], [13], [15], [16]. Similar to previous reports, WBC, IgG and glucose concentrations were included in our model [11], [13], [15]. Failure of transfer of passive immunity, hypoglycemia and leukopenia, which are common findings in critically ill equine neonates and have previously been identified as risk factors for sepsis, and increased mortality, were retained in the FSS [11], [13], [15]. Although high WBC has also been associated with poor outcome in hospitalized foals, leukocytosis had very weak predictive power for survival in our population of foals, and therefore, it was not included in the final model [2], [23].

Clinical signs of poor peripheral perfusion such as prolonged CRT, cold extremities, and injected mucous membranes are frequent and easy to assess variables in critically ill foals [4]. A noteworthy discovery with the FSS was the strong association between cold extremities and increased probability of death. In fact, cold extremities was the factor with the strongest association with mortality, and therefore it was assigned the highest score (2). Previous studies found that neonatal foals with normothermic extremities on admission were 12.2 times more likely to survive [1], or 0.02 times less likely to die than those with cold extremities [5]. Studies in people have shown that the subjective assessment of peripheral perfusion can identify patients with severe organ dysfunction and higher lactate concentrations [24]. A clear advantage of having cold extremities in the scoring system is that it is non-invasive, easy to determine, and enables the clinician to early identify foals with hypoperfusion and increased risk of death.

In addition to cold extremities, hyperlactatemia can be used as a reliable marker of hypoperfusion in foals, horses and people [4], [25]–[27]. Increased blood L-lactate concentrations and reduced L-lactate clearance were associated with severity of disease and increased mortality in critically ill foals [2], [25], [26]. Similarly, in our study foals that survived had lower L-lactate concentrations compared to foals that died. Of interest, L-lactate concentration was not retained in the final model and scoring system. That can be explained in part by the fact that other variables associated with tissue hypoperfusion (cold extremities) had a stronger association with survival than L-lactate concentrations.

Both hypothermia and hyperthermia are frequently observed in critically ill neonatal foals and people [16], [24], [28]. Among newborn children and foals, hypothermia on admission is a significant risk factor for death [16], [24]. Body temperature was also associated with survival in the foals of the study reported here. Although rectal temperature was statistically higher in surviving compared to non-surviving foals, it was not retained in the final model. A similar observation was reported by Hoffman et al, where rectal temperature, heart rate and respiratory rate failed to distinguish surviving from non-surviving foals [14]. Recent research suggest that alterations in peripheral perfusion and microcirculations are independent of clinical signs, and that systemic variables such as temperature or heart rate may not be sensitive enough to reflect changes in peripheral blood flow and to predict the outcome [28]. Of interest, hypothermia was included in the final mathematical model for survival by Furr et al [16]. These differences between studies might be due to variations in severity of disease, selected population of foals, and type of analysis. In the study reported here, foals were unselected, representing a broad spectrum of neonatal diseases, from mild to severe.

Based on previous studies, historical data on maternal diseases and abnormal parturition (dystocia, caesarean section, assisted delivery) were considered clinically important and possible confounders [14]–[16]. These variables were initially forced into our final model, but none were significant, all reduced the fit of the model to the data, indicating that maternal diseases and abnormal foaling have weak predictive power of survival in our population of foals.

To our knowledge this is the first study of this scale in which a large number of sick foals is used to develop a foal survival score that is subsequently validated in a prospective population of foals with a wide range of neonatal diseases, over multiple years. The ideal scoring system should be well calibrated, based on routinely recordable variables, applicable to all patient populations, and have a high level of discrimination [29]. Calibration is the degree of correspondence between the estimated probability produced by the model and the actual observed probability [10], [29]. In our FSS, this was evaluated using a Goodness-of-Fit test. Model discrimination assesses the ability of the scoring system to differentiate between surviving and non-surviving patients based on the predicted survival. In our study, the area under the ROC curve was used to test the discrimination of FSS and was determined to be 0.91. A model with the AUC of ≥0.7 is considered to have adequate discrimination [9], [10], [29].

This study has some limitations that should be acknowledged. While the median age was different between surviving and non-surviving foals and ideally both groups should have been of similar age, age was not retained in the final model, suggesting low predictive power for the outcome. Another important point is that this survival score was developed for foals admitted to three referral centers and its effectiveness in field conditions and other institutions (in different geographic locations) needs to be evaluated. In addition, the FSS was based on data from foals of less than four days of age and its usefulness in older foals needs to be determined.

In conclusion, the survival scoring system developed here can be used as a simple and supplementary tool in helping to determine the likely prognosis for survival of hospitalized neonatal foals on or shortly after hospital admission. Variables included in this survival score can be readily obtained on admission, making it practical in the clinical setting. This score was more effective at predicting survival (positive predictive value = 91%) than at predicting death (negative predictive value = 86%) in foals admitted to the three equine referral hospitals in this study. The survival score developed in this study should not replace clinical judgment and nor should it be used in isolation to make decisions about euthanasia. This information can be used by clinicians and owners to make informed, evidence-based decisions about treatment options for individual neonatal foals based on likely survival.
